# The performance of genetic-constraint metrics varies significantly across the human noncoding genome

**DOI:** 10.64898/2026.01.28.701168

**Published:** 2026-01-28

**Authors:** Peter McHale, Michael E. Goldberg, Aaron R. Quinlan

**Affiliations:** Department of Human Genetics and Utah Center for Genetic Discovery, University of Utah, Salt Lake City, UT 84112, USA

## Abstract

A longstanding goal in human genetics is to prioritize noncoding loci that, when disrupted, lead to developmental disorders and other Mendelian traits. In pursuit of this goal, multiple metrics have been developed to distinguish neutrally evolving sequences from those subjected to purifying selection. These metrics are commonly evaluated genome-wide, e.g., by computing a precision-recall curve on windows tiling the entire noncoding genome. Here, we identify parts of the noncoding genome where these metrics significantly underperform relative to their genome-wide performance due to “bias” in the underlying models of neutral genetic variation and/or a low “signal-to-noise ratio” in the genetic data. The most extreme effects are found for Gnocchi (Chen et al. 2024), the performance of which declines as GC content increases. We suggest annotating constraint scores of noncoding genomic intervals with robust measures of the bias of the corresponding model, allowing users to gauge confidence in those scores.

## Introduction

Computational approaches designed to predict noncoding variant pathogenicity must be improved if they are to have a meaningful impact upon clinical research (Liu et al. 2019). Many of these approaches are based on a simple, but powerful, idea: over the hundreds of millions of years separating species, a single base is likely to have mutated, so the only plausible way to explain why a genomic site is identical between a human and a fish, say, is that whatever mutations occurred there were harmful and eliminated by selection. This deep-time filter is so rigorous that it can detect even weakly constrained elements. Methods like phyloP (Pollard et al. 2010) and GERP (Davydov et al. 2010; Cooper et al. 2005) formalize this idea in a number of ways. (1) They go beyond the vague assertion that a base is “likely to have mutated” by quantifying the expected (neutral) rate of nucleotide substitution between species. (2) They extend the analysis from the comparison of two species to the entire tree of life by accounting for the fact that conservation between, e.g., human and chimp (closely related) is weaker evidence of selection than conservation between human and fish (distantly related). (3) They go beyond a simple binary classification of conservation to answer the question “Is the substitution pattern we see at this site extremely unlikely to have occurred under the neutral model?”

Selection signals derived from sequence conservation are extraordinarily strong and unambiguous but have an Achilles heel: they cannot find genomic elements that are newly constrained in humans, limiting their ability to correctly prioritize noncoding variants. If an element (like an enhancer) becomes functional in the human lineage after the split from chimpanzees, it will not be conserved in chimps, mice, or fish. In scenarios such as these, conservation methods are underpowered (though they can capture elements that have gained function in closely related species, e.g., primates (Boffelli et al. 2003)). The blind spot of conservation-based methods is not small. Rands et al. estimated that only about 25% of the elements under purifying selection in humans are also constrained in mouse (Rands et al. 2014), while, more recently, Huber et al. estimated that most of the noncoding sequence that is functional in human has experienced changes in selection coefficients throughout mammalian evolution (Huber et al. 2020). This process, called “turnover”, had long been suspected from the observation that, as the divergence between mammalian species increases, the predicted amount of pairwise shared functional sequence decreases dramatically (Smith et al. 2004; Meader et al. 2010). Furthermore, most transcription-factor binding events are species-specific, indicative of gain and loss of binding along each lineage (reviewed in (Ponting and Hardison 2011)).

The only way to discover human-specific genetic constraints in the noncoding genome are population-based methods. Pleasingly, early deployment of these methods discovered that regulatory elements located near neural genes gained function in the human lineage (Ward and Kellis 2012; Schrider and Kern 2015), and we expect these methods to become increasingly sensitive for the discovery of gain- and loss-of-function discovery as more human genomes are sequenced. These approaches start with the construction of a model of neutral genetic diversity (e.g., number of SNVs in each of a set of 1kb genomic windows) observed in a healthy human cohort, using features that are known to co-vary with SNV count. Of these, the most well-known feature is the local sequence composition of a given site, whose probability of being polymorphic can increase >10-fold if the sequence is a CpG dinucleotide (Sved and Bird 1990; Bulmer 1986; Schraiber et al. 2024). With a model of this type in hand, one may identify putative signals of negative selection by searching for anomalous regions in the genome where the observed diversity is significantly lower than the neutral model predicts (Chen et al. 2024; Halldorsson et al. 2022; di Iulio et al. 2018). Alternatively, one may construct a model of *all* genetic diversity, including observed diversity impacted by negative selection. In this approach, the model includes a selection parameter, the value of which is estimated for each genomic window (Dukler et al. 2022). The two approaches are intimately related: as Dukler et al. show, the maximum-likelihood estimate of the selection coefficient in the second approach is the ratio of the observed diversity and that expected under neutrality, the comparison of which is the basis of the first approach.

The success of either approach depends upon how well the underlying model of neutrality tracks the observed SNV density across the noncoding genome: if one fails to or inadequately models the neutral mechanisms that, like purifying selection, deplete SNV density, the result is a “biased” model whose predictions of genetic constraint will be flawed. Even if a neutral model can perfectly recapitulate the expected distribution of SNV counts in a locus, one’s ability to predict that the locus is under purifying selection is still limited by how large the “signal” (the depletion of SNV counts due to purifying selection) is relative to “noise” (unavoidable statistical fluctuations in observed SNV counts). Motivated by these concerns, we investigate how existing models’ ability to predict genetic constraint is impacted by variations in model bias and signal-to-noise ratio.

## Results

Consider a recent model of neutral genetic diversity in humans (Chen et al. 2024), which predicts the distribution of the number of SNVs in a given genomic window, aggregated over 76,156 humans, as a function of local sequence context, nucleotide-level DNA methylation, and a comprehensive set of regional genomic features, including GC content and recombination rates. This model yields a score called Gnocchi, denoted *G* below, which evaluates how typical or unusual an observed SNV count is in the context of the window containing the SNVs,

G=R/σ,

where the residual, *R*, is defined by

R=μ−S,

and *S* is the observed SNV count, and *μ* and *σ* are the mean and standard deviation, respectively, of the predicted distribution of SNV counts for that window. A value of Gnocchi near zero indicates that the observed SNV count is close to what one would expect from the neutral model, *μ*. Gnocchi is a *standardized residual*: the residual, *R*, is reported relative to the expected background noise, *σ*. Accordingly, *G* ⪢ 1 indicates the SNV count *S* is not only smaller than *μ*, but also smaller than expected statistical fluctuations below *μ*, raising the possibility (but not guaranteeing) that negative selection is operative in that window.

**Figure 1:**
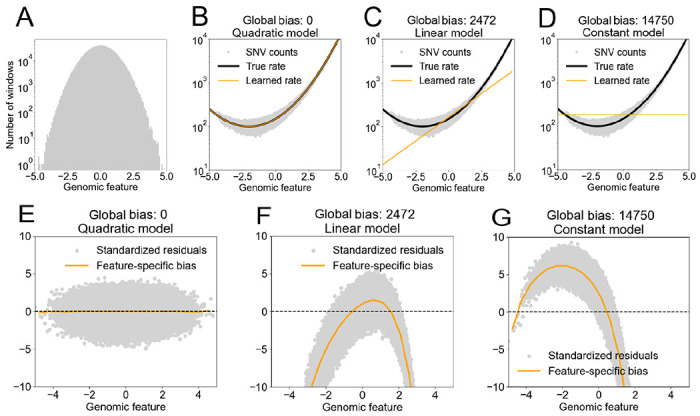
Biased models over- or under-estimate SNV counts, on average, in the tails of the feature distribution. (Top row) Given a mock set of genomic intervals, each with a genomic feature (distributed as shown in A) and a corresponding SNV count (y-axis in B-D), we used Poisson regression to fit three different models mapping the feature to the SNV count (B-D). The quadratic model fits the data perfectly as SNV counts were generated by sampling from a Poisson distribution with a rate parameter that is a quadratic function of the genomic feature (note the concordance of the black line and orange line in B). The linear and constant models of the rate parameter are progressively less complex than the true rate parameter. In these cases, minimization of the sum of negative log likelihoods over all the windows during training forces the learned models to pass through the observed data where they are dense at the expense of deviating systematically from them where they are sparse: compare the grey data points and the orange line in panels C and D. (Bottom row) The degree to which the models over- or under-estimate SNV count can be quantified by the standardized residuals, shown in grey. The average of these data points (orange line) show the feature-dependent biases that emerge when learned models are incomplete. The simulation can be found at the following URL: http://github.com/quinlan-lab/constraint-tools/blob/main/papers/neutral_models_are_biased/9.regression/simulation.4.2.1.interplay-of-SNR-and-bias.ipynb.

Standardized residuals provide information about how the underlying model of neutral diversity is biased. To illustrate how, we developed a simulation consisting of the following sequence of steps. (i) We simulated mutation by random generation of SNVs in a set of mock genomic windows as a function of a mock genomic feature (an example of a real “genomic feature” is “nucleotide content”) distributed normally over windows (Figure 1A). The “generative process” is characterized by a “true rate” at which mutations are generated as a function of the genomic feature (black line in Figure 1B–D), and sampling from a Poisson distribution with that rate (grey dots in Figure 1B–D). (ii) We simulated the development of the model of neutral diversity underlying Gnocchi by estimating the parameters of various models that map the feature value of each window to its SNV count. Like the generative process, the estimated model has its own rate, which we call the “learned rate” (orange line in Figure 1B–D) (iii) We simulated Gnocchi by combining the results of (i) and (ii) to compute a standardized residual (grey dots in Figure 1E–G).

The difference between the estimated model and the generative process is quantified by “global model bias”, defined as the squared difference between the learned rate and the true rate, averaged over all windows (Bishop 2006). We started by using the generated SNVs to estimate a model of the same functional form as the generative process (the “Quadratic model”; Figure 1B). Consequently, after training, the estimated model recapitulated the simulation’s generative process, resulting in zero global model bias (Figure 1B). Corresponding to this lack of bias, the distribution of the standardized residual is randomly scattered around zero independent of genomic feature, indicating that observed and predicted SNV counts agree, on average (Figure 1E). If the estimated model has a simpler functional form than the generative process, such as when the estimated model is linear but the generative process is quadratic, then the global bias becomes non-zero (Figure 1C). Associated with the global bias is a “feature-specific bias”, defined as the mean of the residuals as a function of feature value (orange line in Figure 1F). The feature-specific bias shifts away from zero, particularly in the tails of the distribution of feature values, indicating that the model systematically over- or under-estimates observed genetic diversity. When the model is an even poorer approximation of the generative process, the global bias is even larger (Figure 1D), and the feature-specific bias deviates significantly from zero even in the center of the feature distribution (Figure 1G).

**Figure 2:**
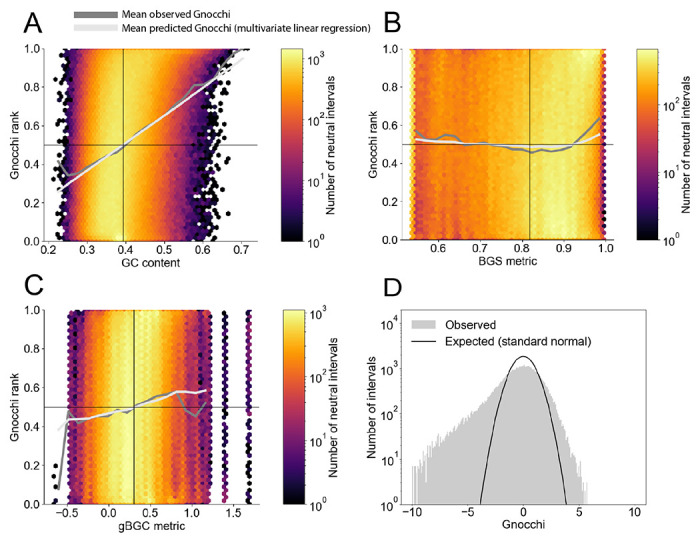
Visualizing the bias of the Chen model of neutral genetic diversity. (A-C) The standardized residuals of the Chen model (Gnocchi) deviate from zero, on average, when the genomic windows they are assessed on exhibit extreme values of GC content, background selection (BGS) or GC-biased gene conversion (gBGC). Since we report the (standardized) rank of Gnocchi on the y-axes, the marginal distribution of Gnocchi (the distribution of Gnocchi over all windows, irrespective of feature value) is uniform with an average value of 0.5 (horizontal black lines). The dark-grey lines are computed by conditioning on each feature bin before computing the mean Gnocchi rank. Note that the slope of the dark-grey lines denote the marginal (not independent) effects of each feature on bias. For example, a position on the dark-grey line in (B) equals the value of Gnocchi conditioned on a specific value of BGS, but integrated over all possible values of GC content and gBGC. The dark-grey lines deviate from the expected value of 0.5, particularly in the tails of the feature distributions (where the heat map is dark). However the dark-grey lines lie at a height of approximately 0.5 when conditioned upon the mean of the feature distributions (vertical black lines). For (B), this implies that the Chen model captures the expected reduction in SNV counts arising from the weak levels of BGS present in the majority of windows, even though the model does not explicitly include BGS, presumably because it covaries with features that the model does include. The co-variation of features with one another allows the linear regression (light-grey line) to track the observed nonlinear behavior (dark-grey line) in (B). (D) Even in the vicinity of mean GC content, the standardized residuals of the Chen model (Gnocchi) exhibit greater variance than expected (from a standard normal distribution), indicating the existence of neutral diversity not captured by the model. GC content was chosen to be within 0.1 * std(GC_content) of the mean GC content, where “std” refers to “standard deviation”. The curve marked “Expected” was constructed by scaling a standard normal distribution according to the bin width of the histogram and the number of intervals. An explanation of why a standard normal distribution is expected using a simplification of the Chen model can be found here: https://github.com/quinlan-lab/constraint-tools/blob/main/papers/neutral_models_are_biased/10.residuals-are-over-dispersed.ipynb.

Motivated by these results, we sought to visualize the bias of the Chen model by plotting the distribution of Gnocchi on a set of 693,270 *putatively neutral* 1kb intervals (Methods) as a function of three features (Figure 2A–C): a measure of nucleotide composition (GC content); a measure of background selection (BGS) computed by (Murphy et al. 2023); and a measure of GC-biased gene conversion (gBGC) computed by (Glémin et al. 2015). We chose GC content as single-nucleotide substitution probabilities have long been known to decline as GC content increases (Fryxell and Moon 2005; Arndt et al. 2005; Mugal and Ellegren 2011). Additionally, there is evidence that GC content at kilobase scale affects the probability of a CpG site mutating, as the deamination process requires local DNA melting, which in turn requires more thermodynamic energy for bonds between G and C nucleotides (Elango et al. 2008). Finally, even the GC content of the local sequence context of a CpG site may correlate with its mutation rate (Chandra and Gao 2025).

Though BGS and gBGC metrics are not explicitly included in the Chen model, they are neutral mechanisms well-known to affect diversity in the human genome (McVicker et al. 2009; Pouyet et al. 2018; Murphy et al. 2023), and have been modeled when inferring natural selection in the context of population genetics, e.g., (Telis et al. 2020). BGS, as a process, reduces variation in regions linked to sequences constrained by purifying selection (for a review see (Charlesworth 2013)). GC-biased gene conversion (reviewed in (Duret and Galtier 2009)) describes how mismatch repair of heteroduplexes formed during meiosis is biased towards GC alleles, and thus against AT alleles, altering their probability of fixation. Therefore allele frequency is also altered in a manner that mimics natural selection though is neutral (Duret and Galtier 2009). As such, like GC content, both BGS and gBGC are neutral processes that lead to reduced genetic diversity which could masquerade as selection to models that fail to account for them.

We observe bias (represented by the deviation of the dark-grey line from *y* = 0.5 in Figure 2A–C) in the Chen model in rare windows that harbor extreme levels of background selection, or GC-biased gene conversion, but particularly those windows with extreme levels of GC content. Note, however, that the lack of bias in the Chen model at intermediate feature levels does not imply that the model cannot be improved there. In fact, we observe that the variance of Gnocchi in that vicinity is larger than what would be expected (Figure 2D), implying that there remains variation in SNV count that the Chen model is unable to capture even at genomic windows whose feature values are observed frequently across the genome.

We next sought to measure how each feature *independently* contributed to the Chen model’s bias. The slopes of the dark-grey lines in Figure 2A–C do not measure these contributions. Instead, these slopes represent not only the direct effect of any given feature (e.g, GC content) on model bias, but also the indirect effect of the complementary features (BGS metric and gBGC) due to correlation among these features. To capture the independent effect of each feature on model bias, we fit a linear model of all features to Gnocchi (Methods). The estimated parameters of the linear model represent the direct effects of the corresponding feature on the bias of the Chen model (Table 1). We find, for example, that the direct effect of the BGS metric on Gnocchi is negative, implying that stronger background selection (smaller values of the BGS metric) is associated with larger values of Gnocchi. This accords with our naive expectation that a model of neutral diversity that does not explicitly account for BGS (as is the case with the Chen model) ought to overestimate diversity where background selection is strong (assuming that BGS does not significantly co-vary with features that the Chen model does include). Interestingly, though a positive value of Gnocchi *erroneously* predicts constraint in the neutral windows subject to strong BGS, it *correctly* predicts constraint nearby, by definition of BGS. Since we standardized all features prior to fitting our linear model to Gnocchi, we may compare the values of the corresponding regression coefficients, revealing that GC content has the largest effect (in magnitude) on model bias: GC content explains almost as much variance in Gnocchi as explained by the three features jointly (compare *ρ*^2^ for one feature vs three features, respectively, in Table 2).

To assess the generality of these results, we repeated our analysis for three other constraint metrics: λ_*s*_ (Dukler et al. 2022), Depletion Rank (Halldorsson et al. 2022) and CDTS (di Iulio et al. 2018) (Methods). The results, presented in Tables 1 and 2, and **Supporting Figure 1**, are similar to those obtained for the Chen model in the sense that all models develop biases as one moves into the tails of the feature distributions, though the details differ. For example, whereas high GC content biases Gnocchi above zero (rank > 0.5), it biases λ_*s*_ below zero (rank < 0.5) (**Supporting Figure 1**). These high-GC-content intervals may be enriched for promoter regions, where the authors of the λ_*s*_ metric observed a similar bias, which they attributed to misspecification of the underlying neutral model there (Dukler et al. 2022). Another difference between the Chen model and the others is that BGS, not GC content, explains most of the bias observed in Depletion Rank and CDTS (e.g., Table 2). It is unclear why, but part of the answer may lie in the fact that the Chen model includes GC content as a factor, but the models underlying Depletion Rank and CDTS do not.

The procedure we adopted to identify the feature that drives model bias is closely related to Gradient Boosting (Friedman 2001), a technique for improving a model by regressing its residuals onto its features, and then adding the resulting “residual model” to the predictions of the original model. Viewed this way, the *ρ*^2^ values reported in Table 2 measure how much an improved modeling of the corresponding features (e.g., via Gradient Boosting) is expected to improve the underlying model of neutral diversity. Comparing the *ρ*^2^ values among features and models leads to two interpretations. First, the largest improvement to the Chen model is expected to come from more complex representations of sequence composition (for which GC content is a surrogate), whereas the largest improvements to the models underlying Depletion Rank and CDTS are expected to come from better representations of BGS. Second, of all the models, the Chen model stands to benefit most from an improved modeling of the three features considered in this study.

The purpose of constraint metrics, such as Gnocchi, is to discover “genetic constraint”, i.e., to distinguish genomic windows that are neutral from those that are under purifying selection. To understand how well this approach is likely to work under various circumstances, we returned to simulation. The standard approach to modeling purifying selection is to use forward-time population genetic simulations, e.g., via stdpopsim (Adrion et al. 2020; Lauterbur et al. 2023; Gower et al. 2025). Though stdpopsim incorporates a host of neutral processes, each of them is expected to contribute to “noise” (unavoidable statistical fluctuations in observed SNV counts in genomic windows), obscuring the patterns we wish to illustrate with our simulations. To minimize this noise, and therefore maximize our ability to recover “signal” (the depletion of SNV counts due solely to purifying selection), we decided instead to return to our simple simulation of neutral diversity (Figure 1A–D), which is stripped of most of the neutral processes included in stdpopsim. We extended the simple simulation to model purifying selection by simply reducing the SNV counts of a random subset of our simulated windows, labeling them “constrained”, and labeling the rest “neutral”. We then built a constraint predictor by thresholding residuals corresponding to each of the models of neutrality depicted in Figure 1B–D: windows with residuals greater than the threshold were predicted to be constrained, whereas those with residuals lower than the threshold were predicted to be neutral. Finally, we assessed the performance of those models by comparing predicted labels with actual labels.

**Figure 3:**
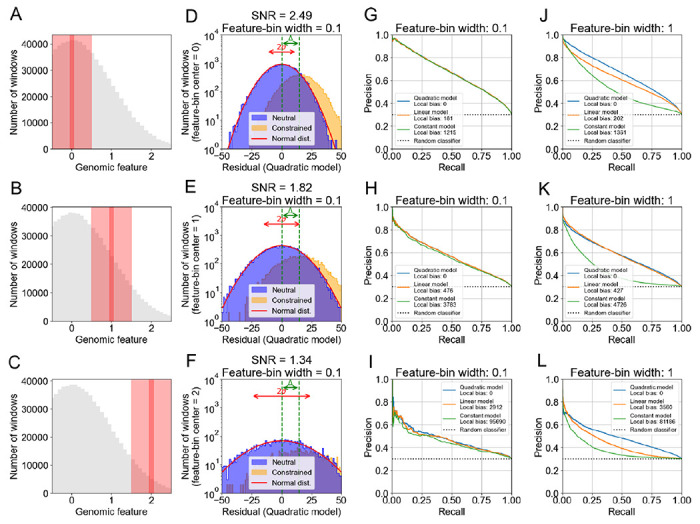
SNR and model bias are both expected to significantly affect the ability to call constraint. The narrow red strips in panels A-C define the subsets of simulated genomic windows that were used to compute panels D-I. Each subset contains neutral (orange) and constrained (blue) windows with residuals distributed as shown in panels D-F (with respect to the unbiased quadratic model). Also shown in those panels are the corresponding values of SNR, computed from the the standard deviation *σ* of the neutral residuals and the depletion Δ of the simulated SNV counts in the constrained windows (Methods). The SNV-count depletion (Δ) and class-imbalance (relative numbers of “constrained” and “neutral” windows) were chosen to be representative of real data (**Supporting Figure 2**). For each window, and each model, we predict that the window is constrained if the residual exceeds a threshold; by varying the threshold we sweep out a PR curve for each model, showing the independent effect of SNR on model performance (panels G-I). To see the independent effect of model bias, compare the PR curves for each model in panels J-L, which correspond to the wide red strips in panels A-C, respectively. “Local bias” is defined similarly to the definition of “Global bias” in Figure 1, but this time using only those genomic windows that comprise the corresponding red strips in panels A-C. The distributions of the constrained windows in panels D-F peak at Δ = 15, which measures the depletion of SNV count that we implemented in the simulation, which can be found here. **Supporting Figure 3** shows similar results when the class distributions are computed using standardized residuals, instead of raw residuals, as shown in panels D-F. **Supporting Figure 4** shows similar results when SNV counts are depleted by an amount proportional to the feature value (i.e., Δ is now feature-dependent), except that now SNR increases (instead of decreasing) as one moves into the right tail of the feature distribution.

Even if a model is unbiased, its performance is limited by the signal-to-noise ratio (SNR): the ratio of the “signal” we are trying to recover (the depletion of SNV counts due to purifying selection) and the unwanted background “noise” we must contend with (unavoidable statistical fluctuations in observed SNV counts). To illustrate this problem and the effect of variations in SNR along the genome, we conditioned on windows with three different values of the genomic feature (Figure 3A–C), and in each case computed the SNR (Figure 3D–F; Methods). Regardless of model bias, performance declines (Figure 3G–I) as SNR declines (reflected in an increasing overlap of the residual distributions of the neutral and constrained windows; Figure 3D–F).

Performance is impacted not only by low SNR, but also by high model bias. To deconvolve these effects, we introduced, for each of the three cases in Figure 3A–C, enough variability in feature values to expose the impact of bias on the performance of the three models, yet not enough to alter the SNR in each case (compare narrow with wide red strips in Figure 3A–C). With this change, not only does performance decline due to declining SNR as found previously (notice the similarity of the green lines in Figure 3J–L with the corresponding green lines in Figure 3G–I), but also because of increasing bias for any given SNR (for each panel of Figure 3J–L, compare the three colored lines).

**Figure 4:**
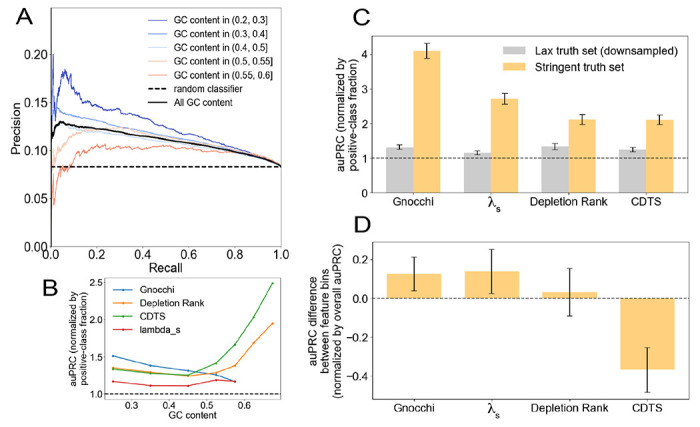
The ability of models of neutral diversity to predict constraint varies with the nature of the genomic windows. (A) The performance of the Chen model at predicting constraint (on a lax truth set defined by whether windows overlap GeneHancer enhancers) can be made to vary significantly by changing the nature of the windows upon which performance is assessed. We varied the Gnocchi threshold to trace out the precision-recall curves. As a benchmark, the performance of a classifier that randomly guesses whether a window is constrained or not has a precision pegged to the fraction of windows that are “positive”, i.e., labeled “constrained” (dashed line; see Methods). To compare performance across GC-content bins, which vary in the proportion of windows labeled “constrained”, we downsampled the bins so that each had an equal fraction of “constrained” windows. (B) Performance varies with GC content in a largely similar way for all constraint metrics, at least for low to intermediate GC content. Performance was defined to be the area under the precision-recall curve, auPRC, normalized by the fraction of examples that are positive, which varies among the lax truth sets. (C) We downsampled the lax truth set so that its size matched that of a stringent truth set, defined by essential genes (Methods), and used 1000 bootstraps of the truth sets to compute a mean and standard deviation of normalized auPRC for each constraint metric and each truth set (without stratifying by genomic features). (D) For each bootstrap sample, we computed the difference in normalized auPRC between bins of high and low values of GC content ((0.4, 0.7) and (0.2, 0.375), respectively), and reported the mean and standard deviation of those differences for each constraint metric relative to the stringent truth set. The results show that GC content is associated with changes in the performance of all constraint metrics relative to the stringent truth set. Code to reproduce C and D can be found here: https://github.com/quinlan-lab/constraint-tools/blob/main/papers/neutral_models_are_biased/11.compare-lax-with-stringent-truth-set.ipynb. In all panels, GC content was assessed in 1kb windows.

Given that model bias and SNR are expected to jointly affect the ability to predict constraint in a way that is impossible to predict without knowledge of that which we are seeking (genetic constraint), we set out to empirically measure performance of Gnocchi as a function of GC content, BGS and gBGC. We designed a simple classifier that predicts that a window is constrained if Gnocchi exceeds a threshold. Since the main purpose of, and main challenge facing, scores such as Gnocchi is to identify constraint in the noncoding genome, an example of which may be a conserved enhancer, we constructed a set of noncoding windows and labeled those that overlap a GeneHancer enhancer (Fishilevich et al. 2017) “constrained”, and those that don’t “neutral” (Methods). Measured against this truth set, Gnocchi clearly does better than random guessing (compare black line and dashed line in Figure 4A), but its performance in GC-rich windows is significantly reduced (red lines); conversely, in GC-poor windows, performance is better than expected (blue lines). Since Gnocchi is biased at low GC content (Figure 2A), and in light of the results in Figure 3, we conjecture that the increased performance at predicting constraint in GC-poor regions is due to an increased SNR. Similar variability in performance was observed as a function of BGS and gBGC (**Supporting Figure 5A, B**). These results illustrate how model bias and/or SNR jointly generate significant variation in ability to discover constraint under the Chen model, depending upon the nature of the windows being interrogated.

Similar trends in performance are observed for the other constraint metrics, with the notable exception of CDTS and Depletion Rank, which increase in performance in GC-rich and/or gBGC-rich regions (Figure 4B, and **Supporting Figure 5C and D**, respectively). Interestingly, these data show that, of all the models, the Chen model has the best *average* performance, i.e., when evaluated at typical values of the features (vertical black lines in Figure 2A–C), even though it has the greatest bias (see *ρ*^2^ in Table 2). Trading significant modeling bias on rare windows for high model performance on common windows has been observed elsewhere (Kathail et al. 2024a).

The number of windows in the truth set that we used to measure performance is large, enabling us to detect performance anomalies that occur deep in the tails of the feature distributions. But that truth set is also lax, as not all windows that overlap a Genehancer enhancer are expected to be highly constrained. For example, 18.4% of the noncoding genome is covered by Genehancer enhancers (Martini et al. 2023), but others have estimated that only 4.51% of the noncoding genome is under human-specific selection (Huber et al. 2020). We therefore identified noncoding windows that we conjectured would be under strong negative selection because they regulate the expression of essential genes (Methods). We combined them with an equal number of noncoding windows not overlapping any enhancer to form a small but stringent truth set. We used it to re-assess the performance of the various constraint metrics at predicting constraint. We observed that overall performance increases on moving from the lax to the stringent truth set, particularly for the Gnocchi and λ_*s*_ metrics (Figure 4C and **Supporting Figure 5E, F**), providing a post-hoc validation of stringency. Even though the stringent truth set is small–making estimates of performance in the tails of the feature distributions noisy–we were nevertheless able to detect associations between performance and features (Figure 4D and **Supporting Figure 5G,H**), as we observed for the lax truth set (Figure 4B and **Supporting Figure 5C,D**).

## Discussion

Sequence conservation leverages the power of deep evolutionary time to provide strong signals of purifying selection, but this approach is limited to the discovery of selection common to a group of species. This forces us to look at human-specific diversity-based methods in order to find human-specific selection. The trade-off is that inference of selection via methods that rely on segregating sites is confounded in a number of ways. (1) Though rare, the fixation of an advantageous allele can drag linked, neutral DNA with it, sweeping away existing variation in that genomic region (Smith and Haigh 1974; Stephan 2019), and naively leading to false-positive calls of purifying selection. (2) During a population bottleneck, rare alleles tend to be lost quickly, before heterozygosity has been eroded, resulting in a transient imbalance between heterozygosity and allele number (Luikart et al. 1998; Amos and Hoffman 2010) that one could falsely interpret as the active removal of deleterious alleles in that interval by purifying selection, if demography is not accounted for. (3) The demographic history of a population can reduce the ability of natural selection to remove weakly deleterious variants (Lohmueller 2014), and so diversity-based methods, which rely on the removal of deleterious variants, will not discover constrained regions harboring those variants. Here, we identify additional factors that confound our interpretation of diversity-based methods for genetic-constraint calling.

We show that current models of neutral genetic diversity are biased, compromising their ability to uncover constraint in the human noncoding genome in rare but important contexts, e.g., high GC content (enriched for promoters) or strong background selection (enriched for proximity to genes), depending upon the model. This mirrors similar findings in the coding genome for a variety of organisms (Yap et al. 2010; Venkat et al. 2018; Ratnakumar et al. 2010).

Similar to the difficulty in capturing edge cases when training machine-learning models to drive cars or answer questions in natural language, models of genetic diversity tend to capture the average behavior seen in a putatively neutral training set, at the expense of incurring large prediction errors on classes of genomic intervals that are rare in that training set. Capturing only average behavior is a blessing when rare events are true anomalies (e.g., constrained windows that are erroneously part of the training set), but a curse when they are not (e.g., truly neutral windows with high GC content).

We find that the model with the highest performance on both the lax and stringent truth set—the Chen model—is also the model with the largest bias, suggesting that bias is a natural tradeoff of improved performance. Interestingly, the direct effect of GC content on bias is larger than that of gBGC and BGS, even though the former was explicitly included in the model whereas the latter were not. Since performance of the Chen model declines with GC content (Figure 4A), and since GC content is positively correlated with Gnocchi (**Supporting Figure 6A,B**), we expect that Gnocchi is most unreliable precisely where it predicts the most constraint. Moreover, given that GC content is auto correlated across the human genome, forming “isochores” (**Supporting Figure 6B**), we expect these false calls of constraint under the Chen model to cluster in genomic space. The relatively high performance of the Chen model (marginalized over all genomic feature values) may result from how comprehensive its set of features is, but it is likely that the way these features, particularly GC content, are included in the model is still not flexible enough to comprehensively capture neutral genetic diversity.

Deep-learning models, such as those used to predict the density of noncoding SNVs (Fang et al. 2022), or their effects on fitness (Benegas et al. 2024; Trevino et al. 2021), do not suffer from such limitations on model complexity. Nevertheless, a deep-learning model mapping genomic features onto SNV density may still fall foul to the type of biases we illustrated here, as recently illustrated for other genomic prediction tasks (Kathail et al. 2024b; Bajwa et al. 2024). To the extent that this failure mode is due to insufficient drive to improve the fit in sparsely populated parts of feature space, a possible solution is to up-weight training examples there, e.g., by weighting a genomic interval in inverse proportion to the number of intervals similar to it (Morcos et al. 2011).

In the meantime, we recommend annotating each genomic interval not only with its constraint score, but also a measure of how biased the score is expected to be in that interval, as a way to gauge confidence in the score. This can be done by grouping genomic intervals by the similarity of their feature vectors (with elements comprising GC content, etc), computing the distribution of constraint scores in each group, and assigning to each window the mean of the constraint-score distribution for the corresponding group.

## Methods

### Provenance of constraint scores

Gnocchi scores for a set of nonoverlapping 1000bp windows (“Chen windows”) were obtained from here (Supplementary Data #2). Windows on the X and Y chromosomes were omitted. Drs Nurdan Kuru and Adam Siepel ran ExtRaINSIGHT (Dukler et al. 2022) on the remaining Chen windows in order to compute λ_*s*_, excluding Chen windows that did not pass the ExtRaINSIGHT filters (private communication). A set of overlapping 500bp windows (“Halldorsson windows”) with Depletion Rank scores (Halldorsson et al. 2022) was obtained from here. A set of overlapping 550bp windows with CDTS scores (“CDTS windows”) (di Iulio et al. 2018) was obtained by private communication with Dr Amalio Telenti (Vir Biotechnology), and their coordinates were lifted over from hg19 to hg38.

### Assignment of genomic feature values to genomic windows

We computed GC content in 1kb intervals centered on the Chen, Halldorsson and CDTS windows using “bedtools nuc”, e.g., as described here and here. We obtained BGS values computed by (Murphy et al. 2023) from here, which we converted to bed format and lifted over from hg19 to hg38 (see here). We obtained the gBGC coefficients on a set of genomic intervals for the European population computed by (Glémin et al. 2015) from here, and lifted over the genomic coordinates from hg18 to hg38, as described here. We assigned BGS values and gBGC coefficients (B_M1star.EUR) to the Chen, Halldorsson and CDTS windows by intersecting them with the bed files of BGS and gBGC, e.g., as shown here and here.

### Construction of the window sets to assess model bias, and the lax truth sets to assess constraint-prediction performance

We filtered out untrustworthy windows, defined as windows that overlap any of the following intervals: gaps in the hg38 genome assembly, Encode “exclude regions” (Amemiya et al. 2019), and regions with insufficient read coverage in Gnomad version 3 (Chen et al. 2024). Exons were computed as shown here and merged using “bedtools merge”. Noncoding windows were defined to be Chen, Halldorsson and CDTS windows that don’t significantly overlap merged exons. Of the noncoding windows, those that don’t significantly overlap Genehancer enhancers (Fishilevich et al. 2017) were labeled “neutral”, and were used to assess model bias. Noncoding windows that do significantly overlap Genehancer enhancers were labeled “constrained”. Together the “neutral” and “constrained” windows comprised the lax truth set used to assess the performance of each of the four constraint metrics.

### Modeling the mapping from genomic features to constraint scores

The code to compute the linear regressions of the constraint scores shown in the heat maps, including the regression coefficients and the proportion of variance explained, reported in the tables, can be found here.

### Computation of window residuals under the Chen model

Each window has a residual, *R*, equal to the difference between the expected SNV count under the Chen model of neutrality, *μ*, and the observed SNV count, *S*. We computed *S* from Gnomad Version 3, using the bash command “constraint-tools predict-germline-model-Nonly --windows ${chen_windows} REQUIRED_ARGUMENTS”, where REQUIRED_ARGUMENTS are specified here. We then used *S*, in conjunction with Gnocchi, *G*, to compute *μ* using the following algorithm:

a=1b=−(2*S+G**2)c=S**2s=sqrt(b**2−4*a*c)sign=1ifG>0else−1μ=(−b+sign*s)/(2*a)

Finally:

R=μ−S.

### Computation of the PR curve for the random classifier

Given a truth set of size *n*, let *π* represent the proportion of windows that are constrained. Let *p* represent the probability that a random classifier predicts that a given window is constrained. Then

$$\text{Precision}=\frac{TP}{TP+FP}=\frac{p\pi n}{p\pi n+p(1-\pi )n}=\pi$$

and

$$\text{Recall}=\frac{TP}{TP+FN}=\frac{p\pi n}{p\pi n+(1-p)\pi n}=p$$

where TP (average number of True Positives) is the average number of windows that are labeled “constrained” in the truth set *and* are predicted to be constrained by the classifier. Similar definitions exist for FP (average number of False Positives) and FN (average number of False Negatives). The formulae for Precision and Recall describe a horizontal line on the Precision-Recall curve (e.g., the dashed line in Figure 4A, traced out as the parameter *p* is varied between 0 and 1) and represents a baseline level of performance that any meaningful constraint predictor ought to exceed.

### Computation of SNR

The signal-to-noise ratio (SNR) is defined as

$$\text{SNR}=\frac{E\left[R_{c}^{2}\right]}{E\left[R_{n}^{2}\right]},$$

where *R_c_* is the random residual of a constrained window, *R_n_* is the random residual of a neutral window, and *E*[·] represents the expectation operator. The numerator is the amplitude of the meaningful signal we are trying to recover (a dip in SNV count due to purifying selection); the denominator is the amplitude of the unwanted background noise we must contend with (unavoidable Poisson fluctuations in SNV count). Squaring the relation

$$R_{c}=R_{n}+\text{Δ},$$

where Δ is the reduction in SNV count induced by purifying selection, we arrive at:

$$R_{c}^{2}=R_{n}^{2}+{\text{Δ}}^{2}+2R_{n}\text{Δ}.$$

Applying the expectation operator, E[⋅] we get:

$$E\left[R_{c}^{2}\right]=\sigma^{2}+{\text{Δ}}^{2},$$

where we used the facts that Δ is constant (if Δ is feature-dependent, then specialize this analysis to a particular bin of that feature), $E\left[R_{n}\right]=0$, and $E\left[R_{n}^{2}\right]=var\left(R_{n}^{2}\right)=\sigma^{2}$. Substituting these expressions for $E\left[R_{c}^{2}\right]$ and $E\left[R_{n}^{2}\right]$ into the definition of SNR, we get

$$\text{SNR}=1+{\left(\frac{\text{Δ}}{\sigma }\right)}^{2}.$$

When Δ ⪡ *σ*, SNR is approximately 1, indicating that the amplitudes of the signal and noise are approximately the same, making it impossible to recover the signal. This is reflected in a flat Precision-Recall (PR) curve (e.g., the dashed line in Figure 4A), which represents a baseline level of performance that any meaningful constraint predictor ought to exceed. We used the SNR formula to compute the numerical values of SNR reported in Figure 3 (and in **Supporting Figure 4**, where Δ is feature-dependent).

### Why PR curves for all models overlap when there is little variability in the mock genomic feature (Figure 3G–I)

Consider a set of simulated windows each with approximately the same value of the mock genomic feature. Under a given model, the predicted SNV count will be approximately the same for all these windows, because these models depend upon only that genomic feature. Now subset the windows into those that are constrained, and those that are not (neutral). The residual distributions for the two classes of windows (constrained and neutral) are just the observed SNV count distributions for the two classes, offset by a constant–the common predicted SNV count. Therefore the overlap of the residual distributions of the two classes is approximately the same for all models, and so all models perform approximately equally.

### Construction of the stringent truth set to assess performance of constraint scores

Chen et al (Chen et al. 2024) obtained a set of highly constrained genes (ClinGen haploinsufficient genes, MGI essential genes, OMIM autosomal dominant genes, LOEUF first-decile genes), linked enhancers to each gene using correlated patterns of histone modifications and gene expression, and assigned the most constrained enhancer to each gene, yielding a set of enhancers that we downloaded from here (Supplementary Data #6). We assigned genomic feature values, and constraint scores, to each of these “essential” enhancers similarly to how we did the assignments for the Chen, Halldorsson and CDTS windows. Finally, we constructed the stringent truth set by combining these “constrained” windows with an equal number of “neutral” windows sampled from the Chen lax truth set. See here.

## Figures and Tables

**Table 1.** The direct effect of each feature on the bias of the neutral models underlying each constraint metric. The regression coefficients may be compared not only within a given model, but also among models, since the features are standardized prior to fitting, and the target variables (ranks) in the fitting process are uniformly distributed between 0 and 1.

constraint score	regression coefficient: feature	Estimated value	Significant?	Standard error
Gnocchi Rank	**BGS**	−0.012969	True	0.000337
	**GC content**	0.081107	True	0.000335
	**gBGC**	0.02247	True	0.000339
λ_s_ Rank	**BGS**	−0.007863	True	0.000361
	**GC content**	−0.04019	True	0.00036
	**gBGC**	0.007627	True	0.000363
Depletion Rank	**BGS**	−0.031909	True	0.000052
	**GC content**	0.014635	True	0.000052
	**gBGC**	0.011318	True	0.000052
CDTS Rank	**BGS**	−0.053583	True	0.000023
	**GC content**	0.009685	True	0.000023
	**gBGC**	−0.006047	True	0.000023

**Table 2.** The proportion of variance in each constraint score that is explained, *ρ*^2^, by all three features considered in this study, and by the single feature with the largest direct effect on bias.

	constraint score	features	*ρ* ^2^
**0**	Gnocchi Rank	[GC content, BGS, gBGC]	0.089556
**1**	Gnocchi Rank	[GC content]	0.082728
**2**	λ_s_ Rank	[GC content, BGS, gBGC]	0.020279
**3**	λ_s_ Rank	[GC content]	0.019076
**4**	Depletion Rank	[GC content, BGS, gBGC]	0.015118
**5**	Depletion Rank	[BGS]	0.010316
**6**	CDTS Rank	[GC content, BGS, gBGC]	0.038306
**7**	CDTS Rank	[BGS]	0.036895
